# Atomistic mechanisms of nonstoichiometry-induced twin boundary structural transformation in titanium dioxide

**DOI:** 10.1038/ncomms8120

**Published:** 2015-05-11

**Authors:** Rong Sun, Zhongchang Wang, Mitsuhiro Saito, Naoya Shibata, Yuichi Ikuhara

**Affiliations:** 1 Institute of Engineering Innovation, The University of Tokyo, 2-11-16, Yayoi, Bunkyo-ku, Tokyo 113-8656, Japan; 2 Advanced Institute for Materials Research, Tohoku University, 2-1-1 Katahira, Aoba-ku, Sendai 980-8577, Japan; 3 Nanostructures Research Laboratory, Japan Fine Ceramics Center, 2-4-1 Mutsuno, Atsuta, Nagoya 456-8587, Japan

## Abstract

Grain boundary (GB) phase transformations often occur in polycrystalline materials while exposed to external stimuli and are universally implicated in substantially affecting their properties, yet atomic-scale knowledge on the transformation process is far from developed. In particular, whether GBs loaded with defects due to treatments can still be conventionally considered as disordered areas with kinetically trapped structure or turn ordered is debated. Here we combine advanced electron microscopy, spectroscopy and first-principles calculations to probe individual TiO_2_ GB subject to different atmosphere, and to demonstrate that stimulated structural defects can self-assemble at GB, forming an ordered structure, which results in GB nonstoichiometry and structural transformations at the atomic scale. Such structural transformation is accompanied with electronic transition at GB. The three-dimensional transformations afford new perspectives on the structural defects at GBs and on the development of strategies to manipulate practically significant GB transformations.

## Abstract (web summary)

Grain boundaries in polycrystalline materials strongly influence their mechanical properties. Here, the authors investigate polycrystalline TiO_2_ by high-resolution electron microscopy and observe that structural defects form ordered structures at grain boundaries influencing their properties.

## Graphical abstract

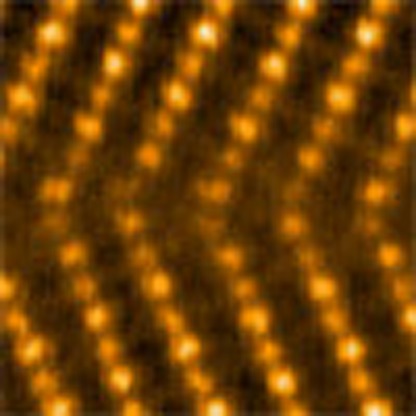

Naturally occurring and artificially fabricated materials are usually of polycrystalline character, constituting numerous single-crystalline grains bonded to one another across the interfaces called grain boundaries (GBs). In general, these intercrystalline defects and their interaction with atomic defects and impurities govern the properties of many materials^1^,^2^. Such impacts shall be particularly pronounced in the materials undergone external stimuli, (for example, heat and atmosphere) where point defects are stimulated and GBs can serve as effective sinks for them possibly giving rise to GB structure modification. Such GB transformation is of significant fundamental interest and practical importance as it might markedly affect the mechanical, electrical and quantum electron properties of a broad range of materials and hence many of their applications^3^. However, it remains a nontrivial task to investigate the transformation of an individual GB at the atomic scale because spatial resolution of all the atoms within the two-dimensional defective areas inside materials still poses a significant challenge, thereby limiting an atomistic understanding of the structure–property interplay. Transmission electron microscopy (TEM) is, in principle, able to probe atomic structures of GBs and their transformation, especially in the high-angle annular dark field (HAADF) mode. In addition, the aberration-correction optics can advance point-to-point resolution greatly^4^,^5^,^6^,^7^,^8^. However, for those ceramics comprising atoms with a vastly different atomic number *Z*, directly imaging the atoms with a low *Z* via the HAADF mode is still difficult because they only weakly scatter electrons, thereby hindering the determination of the exact site of every individual entity at the GB.

To date, much of the knowledge on GB transformation has been developed by theoretical calculations especially for metals. For example, Frolov *et al*.^9^ proposed a computational methodology, which allows variations in atomic density inside a GB using the empirical potentials, and predicted multiple GB phases in face-centred cubic metals. These efforts are, however, much complicated for ceramics due to the availability of potentials and also because one has to consider the charge issues and electrostatic driving force, which may induce segregation of point defects to GBs. Titania, which is a wide-band-gap (∼3 eV) semiconductor and finds applications in a wide variety of technological fields, including catalysts, photocatalysts, solar cells, gas sensors and waste remediations^10^,^11^, represents such a case. Despite that in many circumstances, GBs are known or suspected to affect the functionalities of TiO_2_ (ref. 12), resolving the exact sites of oxygen atoms comprising the GB is still challenging^13^,^14^. The direct determination of oxygen atoms is relevant because the oxygen atoms have a strong electronegativity and how they fill GB sites markedly affects TiO_2_ functionality via either binding the free electrons resulting in a loss of electrical conductivity and catalytic activity or forming vacancies leading to an enhanced ionic conductivity^15^,^16^.

Here combining aberration-corrected HAADF^17^, annular bright-field (ABF)^18^,^19^ scanning TEM (STEM) and first-principles calculations, we obtain a direct imaging of all the atoms in a commonly occurring Σ3 GB (Σ stands for degree of geometrical coincidence at a GB) of TiO_2_ with atomic resolution and electronic sensitivity, and offer evidence to GB structural transformations in TiO_2_ induced by heat and atmosphere treatment, a usual way to tailor properties of TiO_2_.

## Results

### Microstructure of the bicrystal

**Figure 1:**
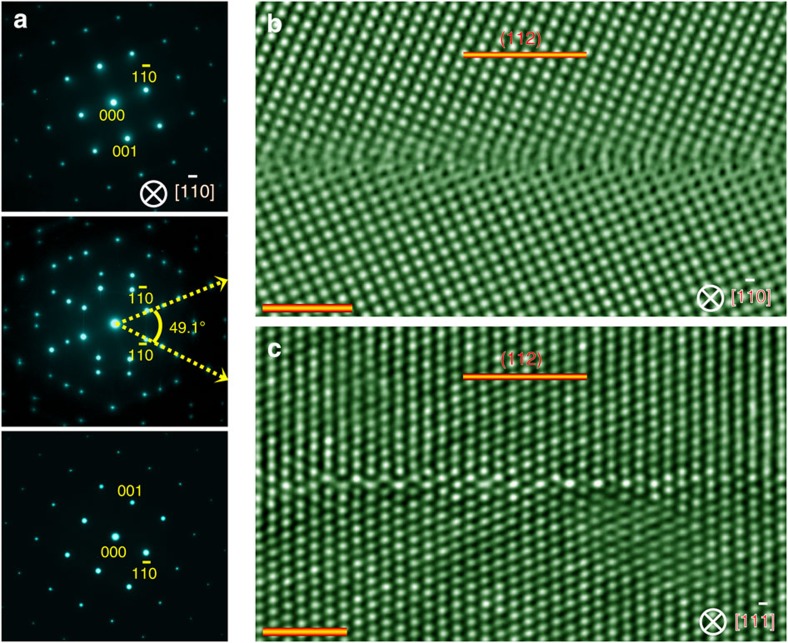
Microscopic analysis of as-prepared bicrystals. (**a**) Selected-area diffraction patterns taken from 
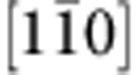
 direction at the upper crystal (top), GB (middle) and lower crystal (bottom). Symmetry is seen in the pattern, indicating a perfect joining of the two single crystals. The misfit tilt angle is determined to be ∼0.04°. (**b**,**c**) A representative cross-sectional high-resolution TEM image of the Σ3 *o*-GB taken along 
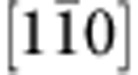
 (**b**) and 
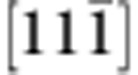
 (**c**) directions. The GB is atomically flat. Scale bar, 2 nm.

In general, polycrystalline materials contain a wide variety of GBs (highly symmetrical and random GBs) that are affected by numerous factors, such as grain orientation, growth history and processing condition^20^. To limit the number of degree of freedom^21^ associated with the random GBs so as to provide a realistic opportunity to explore individual GBs, we apply the bicrystal technique to fabricate a Σ3(112) 
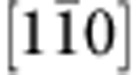
 symmetric tilt GB of TiO_2_ (named *o*-GB) by solid-state diffusion bonding of two single crystals (Supplementary Fig. 1)^22^,^23^. It is noteworthy that as an initial step to probe how an individual GB is affected by external stimuli, we selected a highly symmetrical twin boundary rather than the random GBs in that it raises the likelihood to directly resolve all the entities at the GB, thereby allowing us to gain a deeper understanding of how GBs transform at the atomic scale. Analysis of the selected-area diffraction pattern confirms the Σ3 orientation relationship (Fig. 1a, Supplementary Fig. 2), and the TEM and STEM imaging along two orthogonal directions 
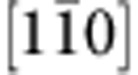
 and 
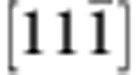
 shows an exclusive contact of the two crystals at the GB (Supplementary Fig. 3). To extract the atomic information, we obtain aberration-corrected high-resolution TEM images (Fig. 1b,c), which reveal that the GB is atomically abrupt and periodic. In addition, there occur periodic structural units on the GB mirror plane.

### Atomic-scale imaging of the as-prepared GB

**Figure 2:**
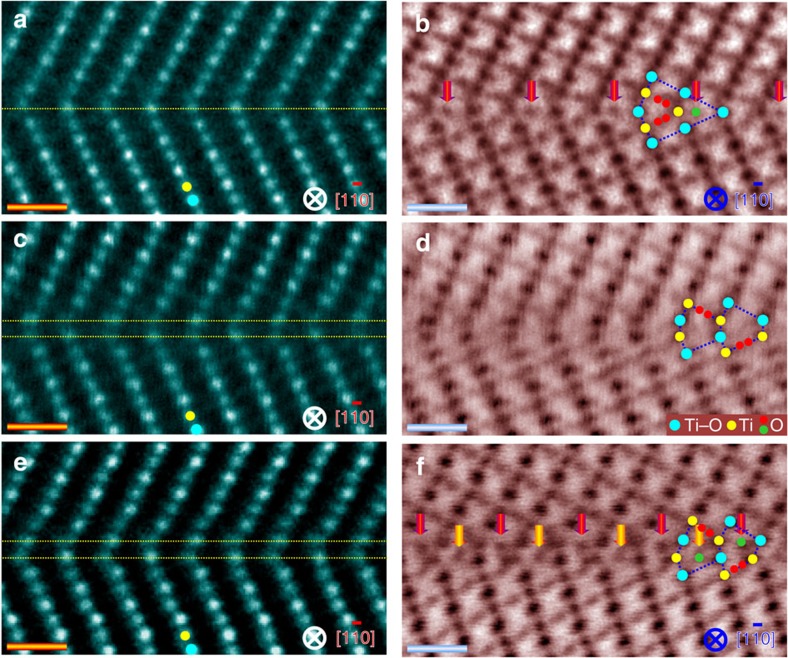
Direct atomic-resolution imaging of the GBs. (**a**,**b**) Atom-resolved HAADF (**a**) and ABF (**b**) STEM images for the *o*-GB viewed along 
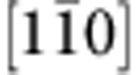
 direction. The light blue and yellow circles indicate the Ti-O and the Ti-only atomic columns. The O atoms are marked in green, while those in bulk are highlighted in red. (**c**,**d**) HAADF (**c**) and ABF (**d**) images for the *r*-GB viewed along 
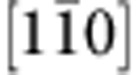
 direction. (**e**,**f**) HAADF (**e**) and ABF (**f**) images for the *v*-GB. The HAADF images are recorded simultaneously with ABF STEM images. A rigid structural unit is superimposed on the ABF images. The spots with a weak image contrast (O columns) are highlighted by arrows. A periodicity is seen along the boundary in the both projections. Scale bar, 5 Å.

To identify the chemistry of the GB, we perform electron energy-loss spectroscopy (EELS) analyses of both bulk and GB area over a broad energy range, as shown in Supplementary Fig. 4 (ref. 24). Only the signatures of Ti-L_2,3_ and O K edges are detected in the entire spectrum at GB, which implies that the *o*-GB may not contain a substantial amount of impurities. To provide atomic details, we show in Fig. 2a HAADF STEM image viewed from 
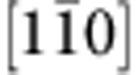
 direction, revealing alternating spots exactly on the mirror plane. Since image intensity of an atomic column in a HAADF STEM mode is roughly proportional to *Z*^1.7^, the contrast in this image is brighter for either heavier atoms or for the larger atomic density in the atomic column^25^. This could, in principle, allows us to identify the chemical nature of the atomic columns in the HAADF images. In this sense, the light spots represent pure Ti columns, while the brightest ones represent Ti-O mixed columns because the Ti-O mixed columns have a much larger atomic density along individual columns. This indicates that the monolayer on mirror plane comprises of alternating Ti and Ti-O atomic columns. We also obtain a HAADF STEM image from 
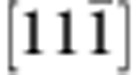
 direction, as shown in Supplementary Fig. 5a, which reveals the same periodicity at the GB as that in bulk. The much lighter O atoms are not scattered strongly enough to be visualized, rendering the HAADF image incomplete.

To directly resolve all the atoms at the GB, we apply the ABF STEM imaging technique^18^ to observe the *o*-GB, as shown in Fig. 2b. The ABF STEM approach has been proven to allow a simultaneous imaging of light and heavy atoms with a good signal-to-noise ratio, robustly over a range of sample thickness^18^. Apart from conveying the basic structural information as that in the HAADF, the ABF STEM image, in which dark spots represent atomic columns, reveals additional spots with weaker image contrast aligned periodically in the open space on the mirror plane (Fig. 2b, arrow). In light of the weak image contrast and the comparison of the HAADF (Fig. 2a) with ABF images (Fig. 2b), these extra spots are identified as O columns. To offer more details on the GB structure, we also show ABF STEM image from the orthogonal 
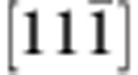
 direction, uncovering extra periodic spots on the mirror plane (Supplementary Fig. 5b, arrows).

### Impact of external treatment on GB structure

To explore how individual GB behaves under external stimuli, we perform independent annealing of the bicrystal in reduced atmosphere (*r*-GB) and in vacuum (*v*-GB). An EELS analysis of *r*-GB reveals the presence of Ti and O signals solely in its detection limit, as shown in Supplementary Fig. 4. Figure 2c,d shows HAADF and ABF STEM images of *r*-GB along 
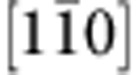
 projection, which reveal that the *r*-GB undergoes a transformation, yet remains ordered within the original orientation relationships. In contrast to the monolayer exactly on the mirror plane at the *o*-GB, a bilayer forms at the *r*-GB, which comprises of Ti and Ti-O atomic columns that alternate within an inverse mirror symmetry (Fig. 2c). Further ABF STEM image reveals an almost free space inside each structural unit (Fig. 2d), that is, oxygen is heavily deficient at the GB. Such structural change can be attributed to the formation of amounts of O vacancies induced by annealing, which are segregated to the GB, self-assembling to form an ordered structure. This structure is also supported by the ABF STEM imaging from 
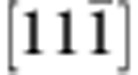
 direction, which reveals periodic zigzag spots with weak image contrast at the GB (Supplementary Fig. 5d, arrows), albeit that the *r*-GB exhibits an identical HAADF STEM image to the *o*-GB along this projection.

To gain insights into how treatment condition can impact GB transformation, we show in Fig. 2e HAADF STEM image for the *v*-GB. While maintaining the same orientation relations, the *v*-GB shows a vastly different structure from the *o*- and *r*-GBs: the mirror symmetry is now broken with the Ti (weaker) and Ti-O (brighter) columns at the GB shifted from each other along the GB, that is, the two layers on the mirror plane (cusps of kites) are arranged in a zigzag manner. Further EELS analysis of the boundary region reveals the presence of Ti and O signals (Supplementary Fig. 4), implying that the intrinsic oxygen vacancy may account for the GB transformation. In contrast to the presence of free spaces at the *r*-GB, an oxygen atom is trapped inside each structural unit (Fig. 2f, arrow) and is zigzag aligned along the GB. We also obtain HAADF and ABF STEM images taken from 
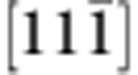
 projection, revealing a zigzag distribution of O at the GB (Supplementary Fig. 5e,f). These results indicate that a certain amount of oxygen vacancies forms at GB, which are self-assembled to form an ordered structure, giving rise to GB transformation.

## Discussion

To provide a deeper understanding on the GB structural transformations, we conducted density-functional-theory^26^ calculations using GGA (generalized gradient approximation)^27^ plus the *U*-method. To systematically search the GB geometry, we constructed two starting models within the mirror symmetry: one has a monolayer (Supplementary Fig. 6a) and the other has a bilayer at the GB (Supplementary Fig. 6e). These two models contain all the possible O occupations at the GB in consideration of the space filling, thus enabling to systematically introduce charge-compensating O vacancy. In either case, we first consider all the likely lattice sites around the GB to introduce an O vacancy once at a time and calculate the segregation energy (*E*_seg_) using the relation *E*_seg_=(*E*_GB_−*E*_BR_), where *E*_GB_ and *E*_BR_ are total energies of the optimized supercells with a single O vacancy at the GB and in bulk, respectively. The model with the lowest *E*_seg_ is then used to further introduce all likely O vacancies around GB once at a time (Supplementary Fig. 6), followed by a calculation of the *E*_seg_ for the relaxed supercells with a single O vacancy (Supplementary Table 1). Such search process is repeated until all the likely O atoms at GB are removed, which produces a series of stable GB structures with different densities of O vacancy at GB.

**Figure 3:**
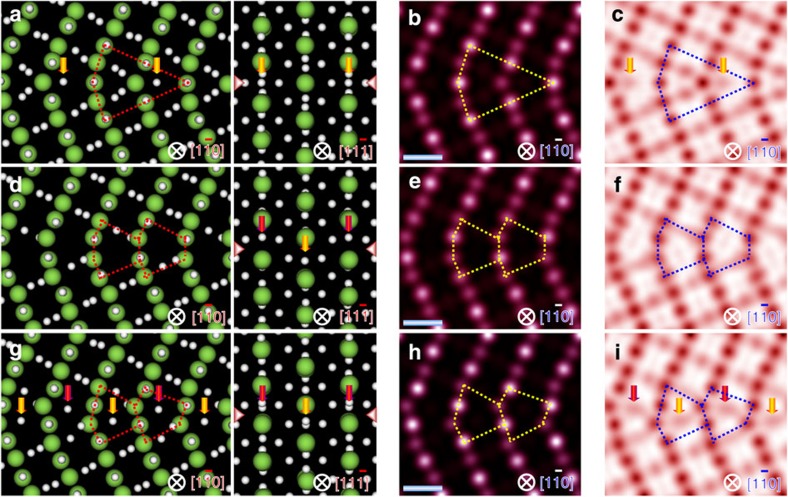
Identification of ordered-defect-induced GB transformation. (**a**) A periodic supercell adopted to model the *o*-GB viewed from 
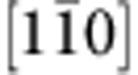
 (left) and 
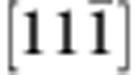
 (right) direction. The larger balls represent Ti atoms and the smaller ones O atoms. The GB structural unit is highlighted by a polygon. The spots with a weak contrast in the ABF STEM images are recognized as O columns, as marked by arrows. (**b**,**c**) Simulated HAADF (**b**) and ABF (**c**) STEM images using the determined *o*-GB atomic model viewed from the 
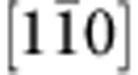
 direction. (**d**–**f**) Atomistic model (**d**), simulated HAADF (**e**) and ABF (**f**) images of the *r*-GB. (**g**–**i**) Model (**g**), simulated HAADF (**h**) and ABF (**i**) STEM images of the *v*-GB. A good match is acquired between the experimental and the simulated images in both the projections, in further support of the GB structural transformations. Scale bar, 3 Å.

Of all the stable structures, we identify three atomic models (Fig. 3a,d,g) that match correspondingly with the experimental images in the three cases. Upon a further calculation of stoichiometry for every GB configuration (Supplementary Discussion), we demonstrate that the *o*-GB, *v*-GB and *r*-GB are oxygen that are locally over-stoichiometric, stoichiometric and under-stoichiometric, respectively, indicating that nonstoichiometry accounts for the GB transformation. Upon a closer inspection of these models, we offer direct support to the observation of O exactly on the mirror plane at the *o*-GB (Fig. 3a), the free space at *r*-GB (Fig. 3d) and the trapping of an O atom in individual structural unit at the *v*-GB (Fig. 3g). As a further confirmation, we simulated the HAADF and ABF STEM images using the determined GB models^28^ and compared them (Fig. 3, Supplementary Fig. 7) with their respective experimental counterparts (Fig. 2, Supplementary Fig. 5). We found a good agreement in both the projections particularly in terms of the oxygen distribution, in further support of the GB transformation at the atomic scale as a consequence of the external stimuli.

**Figure 4:**
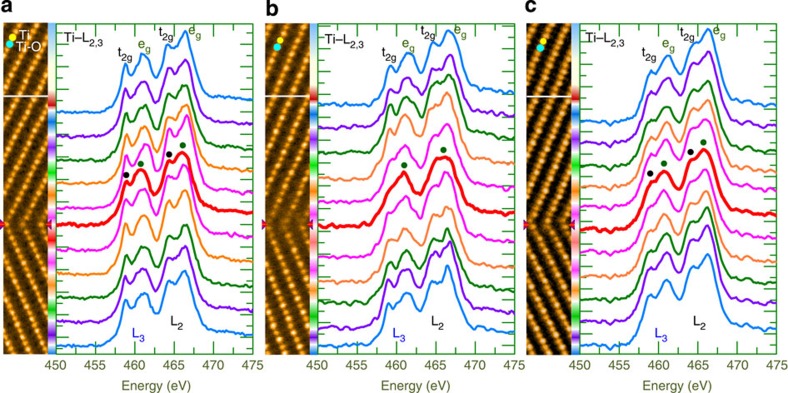
Electronic shift at GB. (**a**–**c**) High-resolution HAADF STEM image (left) and corresponding EELS profiles of the Ti-L_2,3_ edge (right) recorded across the *o*-GB (**a**), *r*-GB (**b**) and *v*-GB (**c**). The GB electronic property is modified as a consequence of the structural transformation.

To probe the electronic impact of GB transformation, we perform EELS spectrum analyses of Ti-L_2,3_ edge for the three types of GBs, as shown in Fig. 4. The Ti-L_2,3_ EELS spectra obtained in the region away from the GB consist mainly of four peaks (two doublets), which comprise Ti-L_3_ edge at the lower energy loss and Ti-L_2_ edge at the higher energy loss, a characteristic of having a valency of +4 for the Ti away from the GB. Such a valence state holds for the Ti ions at the *o*-GB, as the four peaks are visibly split (marked by dots in Fig. 4a). However, the Ti-L_2,3_ EELS spectrum for the Ti atoms at the *r*-GB exhibit a broader profile (marked by dots in Fig. 4b), suggesting that they are of a mixed valence state of +3/+4. On the contrary, the splitting of four peaks is visible in the EELS spectrum of Ti-L_2,3_ edge for the Ti atoms at the *v*-GB (marked by dots in Fig. 4c), indicating that they have a valence state close to +4. Such shift in electronic states points to the existence of novel impacts associated with the defect-GB interactions by treatment and highlights the importance in atomic-resolution imaging of GB transformation.

**Figure 5:**
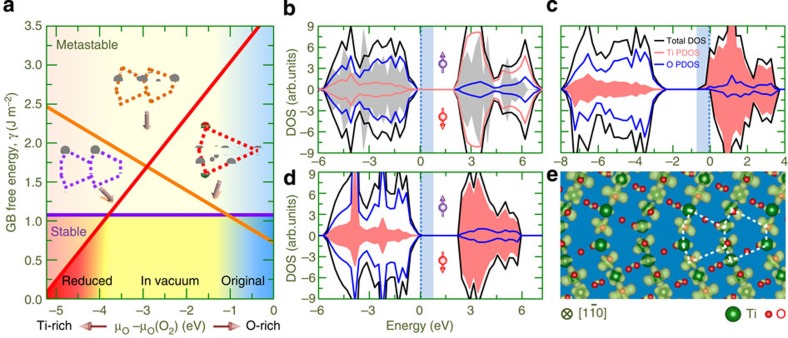
Relative stability and electronic properties of GBs. (**a**) Calculated free energy as a function of O chemical potential (*μ*_O_) for the three GBs. The Ti-rich and O-rich ends of the chemical potential scale correspond to cases where TiO_2_ is in equilibrium with the metallic Ti and gaseous O_2_, respectively. The orange, red and purple lines denote *o*-GB, *r*-GB and *v*-GB, respectively. The inset shows the structural transformations induced by the atmosphere treatment. (**b**–**d**) Total DOS and PDOS plots of Ti and O atom contribution for the *o*-GB (**b**), *r*-GB (**c**) and *v*-GB (**d**). Total DOS of bulk TiO_2_ is given in **b** as a grey background and the Ti PDOSs are highlighted by shading in **c** and **d**. The Fermi level is set to zero and represented by vertical lines. (**e**) Isosurface along 
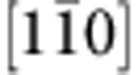
 plane for the *r*-GB, which is integrated in an energy window (*E*_F_−0.7 eV, *E*_F_).

To shed light on the conditions at which the predicted GB models are stable, we further calculate Gibbs free energy (*γ*) as a function of O chemical potential (*μ*_O_) (Supplementary Discussion), as shown in Fig. 5a. We first note that the *o*-GB shows the lowest *γ* at O-rich condition, consistent with its O over-stoichiometric nature. The *r*-GB, on the other hand, exhibits the lowest *γ* at Ti-rich condition, in line with its O under-stoichiometric nature. The *v*-GB treated under a medium O partial pressure exhibits the lowest *γ* in intermediate range of *μ*_O_, in agreement with its O stoichiometric nature. The relative stability reflects qualitatively the adopted experimental conditions, validating application of these models to describe the observed GBs.

To gain more insights into the physical mechanism of the electronic property shift and the quantum nature of the conducting states, we show in Fig. 5b–e electronic structures of the three GBs calculated using their fully optimized geometries. Our calculations reproduce unambiguously the electronic property shift (Fig. 4): for the *o*-GB, Fermi level (*E*_F_) lies in a gap, splitting the filled O 2*p* orbitals from the unfilled Ti-3*d* levels; for the *r*-GB, the low-lying Ti-3*d* bands dominate density of states (DOS) around *E*_F_ and a ferromagnetic alignment of spins is preferred (Fig. 5c); for the *v*-GB, the *E*_F_ lies in a gap between states (Fig. 5d). Further analysis of orbital contribution uncovers that the filled states around *E*_F_ in the *r*-GB case come mainly from *d*_yz_, *d*x^2^-y^2^ and *d*z^2^ orbitals (Supplementary Fig. 8), as also reflected in the charge density isosurface (Fig. 5e). On a close inspection of the band structure, we find that the two lowest-lying filled 3*d* conduction bands in the *r*-GB case show no discernible dispersion along the Γ-X and Γ-*Z*, yet a strong dispersion along the Γ-Y, suggesting that the *r*-GB exhibits a spin-polarized quasi-one-dimensional character (Supplementary Fig. 9). The predicted shift in the electrical conductivity and magnetism at the GB may be important for realizing functional devices by GB engineering.

Direct atomic-scale observation of nonstoichiometry-driven ordered GB transformation through post treatments advances greatly our understanding on GB behaviours in ceramics in that GBs markedly loaded with defects tend to be viewed as disordered areas. Previous studies have uncovered the existence of interphases or pre-melting transitions at the GBs in metallic alloys and intergranular amorphous films in ceramics^29^,^30^, but we demonstrate, by identifying each individual entities at the GBs, that GBs can absorb a large amount of structural defects, which self-assemble to form anion ordering, driving GB transformation, despite that the segregation of defects either at GBs or on surfaces has been reported^31^,^32^,^33^,^34^,^35^,^36^. The finding of the ordered structures takes on practical relevance in polycrystalline TiO_2_, where hydrogenation or treatment is often used to enhance its functionality (for example, catalytic or photocatalytic). The combined method to directly resolve all sublattices in individually treated GBs should be applicable to other complicated GBs in a wide range of ceramics.

## Methods

### Sample preparation and characterization

The pristine Σ3(112) 
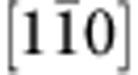
 symmetric tilt GB of TiO_2_ was prepared by the bicrystal technique via solid-state diffusion bonding of the two rutile TiO_2_ single crystals of high purity in air (Supplementary Discussion)^22^. Specimens for the TEM and STEM imaging were prepared by cutting, grinding, dimpling and argon ion-beam thinning process. Some of the specimens were then independently annealed at 1,073 K for 4 h at a reduced atmosphere of Ar (95%) and H_2_ (5%) and in vacuum (2 × 10^–3^ Pa). The selected-area diffraction pattern, TEM and high-resolution TEM images were taken using the JEOL JEM-2010F and aberration-corrected FEI TITAN80-300 electron microscopes. HAADF and ABF images were taken with the 200-kV STEM (ARM-200FC, JEOL) equipped with a probe corrector (CEOS Gmbh), which offers an unprecedented opportunity to probe structures with a sub-ångström resolution. For the HAADF STEM imaging, we adopted a probe convergence angle of ∼22 mrad and a detector with inner semi-angle of >60 mrad. The ABF STEM images were taken with a detector of 6–25 mrad, and the EELS was recorded using a Gatan Enfina system equipped on the STEM with an energy resolution (full-width of half-maximum) of ∼0.5 eV. Image simulations were carried out using the WinHREM program (HREM Research Inc.), which was based on the multislice method^28^. The absorption of the thermal diffuse scattering was taken into account for each element involved.

### Quantum-mechanical calculations

Calculations of GB energies and electronic structures were conducted using the Vienna *ab initio* simulation package^26^ within the framework of density-functional-theory. We applied projector augmented wave^37^ method with 4 × 4 × 1 *k*-point grids and a cutoff energy of 400 eV, which enabled an accurate description of both the atomic and electronic structures of GBs. The GGA+*U*-method was applied with *U*=4.5 and *J*=1.0 eV for Ti-*3d* orbitals, which had been demonstrated to enable an accurate prediction of both the atomic and electronic structures of TiO_2_. The GBs were modelled by periodic supercells with a dimension of 6.55 × 7.20 × 35.89 Å and further doubled along the boundary direction to examine the effect of supercell size. We found that size of the supercell imposed no fundamental impact on the electronic structures of the GBs. The *o*-GB, *v*-GB and *r*-GB supercells had 98 O and 48 Ti atoms, 96 O and 48 Ti atoms, and 94 O and 48 Ti atoms, respectively. It was noteworthy that each supercell had two equivalent GBs^38^,^39^, and the two equivalent oxygen atoms in the two GBs were removed simultaneously while taking into account the oxygen vacancy effect. All atoms in the supercells were fully relaxed at each step when an oxygen vacancy was introduced until the magnitude of force on each atom fell below 0.05 eV per Å. The calculated lattice parameters for the rutile TiO_2_ bulk were *a*=4.632 Å and *c*=2.983 Å, consistent with the experimental values^40^.

## Author contributions

R.S. designed and conducted the experiments and wrote the paper. Z.W. designed the experiments, performed simulations and supported to write the paper. M.S. assisted the experiments and data analysis. N.S. supported the experiments and discussed the results. Y.I. discussed the results and directed the entire study. All the authors read and commented on the paper.

## Additional information

**How to cite this article:** Sun, R. *et al*. Atomistic mechanisms of nonstoichiometry-induced twin boundary structural transformation in titanium dioxide. *Nat. Commun.* 6:7120 doi: 10.1038/ncomms8120 (2015).

## Supplementary Material

Supplementary Information Supplementary Figures 1-9, Supplementary Table 1, Supplementary Discussion and Supplementary References
