# Supplementary material for: Atomistic mechanisms of nonstoichiometry-induced twin boundary structural transformation in titanium dioxide
Source: Nat Commun. 2015 May 11;6:7120. doi: 10.1038/ncomms8120 (PMC4432645; doi:10.1038/ncomms8120)
Supplement: Supplementary Information — Supplementary Figures 1-9, Supplementary Table 1, Supplementary Discussion and Supplementary References [file ncomms8120-s1.pdf]

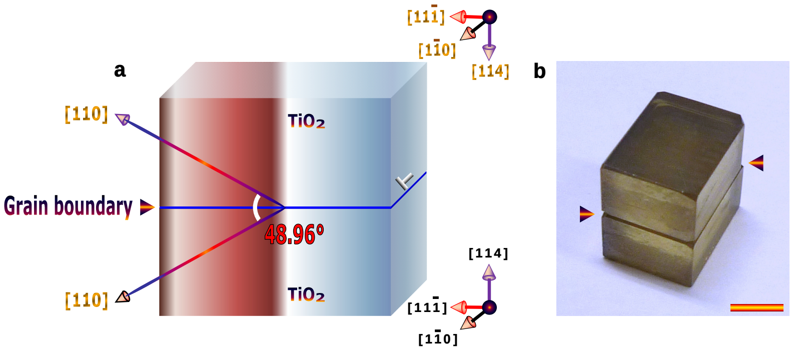

Supplementary Figure 1 | Orientation relationships and photograph of the bicrystal. **a,** Sketch of the TiO_2 bicrystal with the bi-crystallographic relationships $(112)[1\bar{1}0]_{upper} \parallel (112)[1\bar{1}0]_{lower}$ between the upper and lower single crystals. **b,** A real photograph of the final bicrystal block. The arrows point to the boundary. Scale bar, 5 mm.

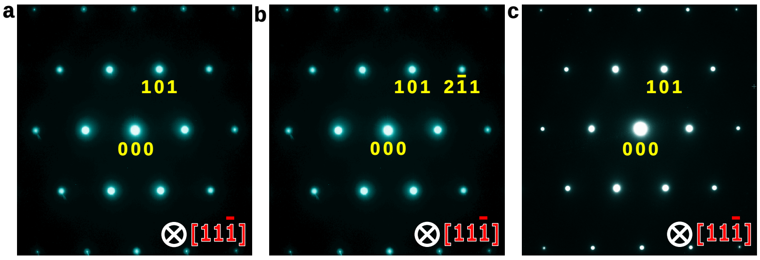

Supplementary Figure 2 | Confirmation of structural symmetry. a–c, Selected-area diffraction pattern (SADP) taken at the upper crystal (**a**), GB area (**b**), and lower crystal (**c**) viewed from [11$\bar{1}$] direction for the (112)[1$\bar{1}$0] *o*-GB. Symmetry is seen, confirming a perfect joining of the two single crystals within the pre-designated orientation relation.

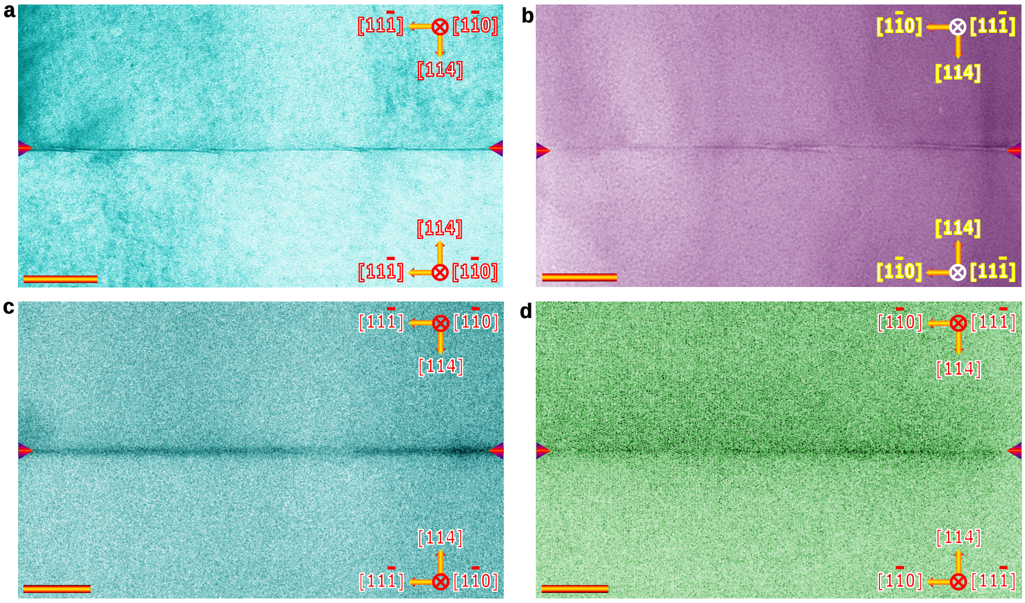

Supplementary Figure 3 | Low-magnification TEM and STEM images of the GB. a,b, Low-magnification TEM images of boundary area in the as-prepared (112)$[1\bar{1}0]$ bicrystal viewed along $[1\bar{1}0]$ (**a**) and $[11\bar{1}]$ (**b**) direction. Scale bar, 50 nm. **c,d,** Low-magnification ADF STEM image of the (112)$[1\bar{1}0]$ *o*-GB, viewed along the $[1\bar{1}0]$ (**c**) and $[11\bar{1}]$ (**d**) direction. The GB position is indicated by arrows. Scale bar, 30 nm.

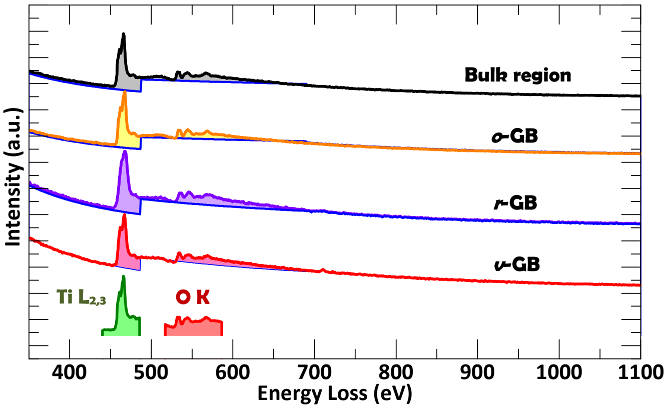

Supplementary Figure 4 | Chemical analysis. EELS taken around the three species of GBs and in bulk (away from GB) in a broad energy-loss range containing Ti $L_{2,3}$ and O K edges. Only the Ti and O signals are detected in the spectra independent of the treatment at different atmosphere. The spectra with background subtracted are given at the bottom.

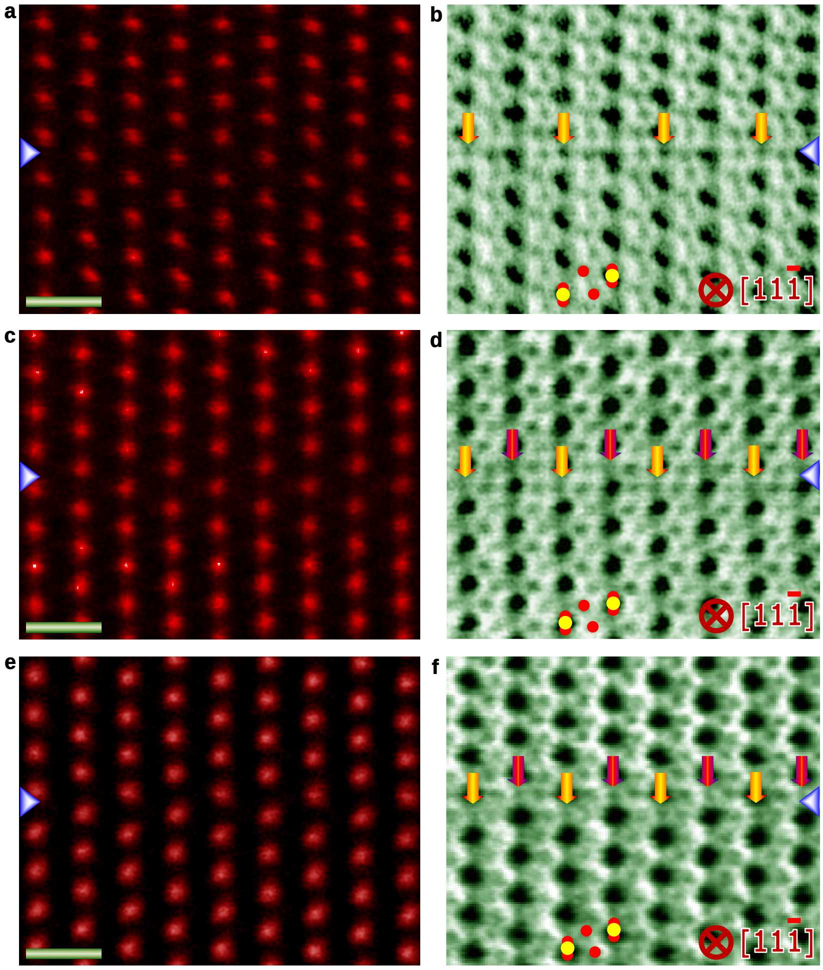

Supplementary Figure 5 | Direct atomic-resolution imaging. a,b, Atomic-resolution HAADF (**a**) and ABF (**b**) STEM images for the *o*-GB viewed from the orthogonal [11$\bar{1}$] projection. **c,d,** HAADF (**c**) and ABF (**d**) STEM images for the *r*-GB (**h**). **e,f,** HAADF (**e**) and ABF (**f**) STEM images for the *v*-GB. The spots with weak image contrast (O column) are marked by arrows. Scale bar, 5 Å.

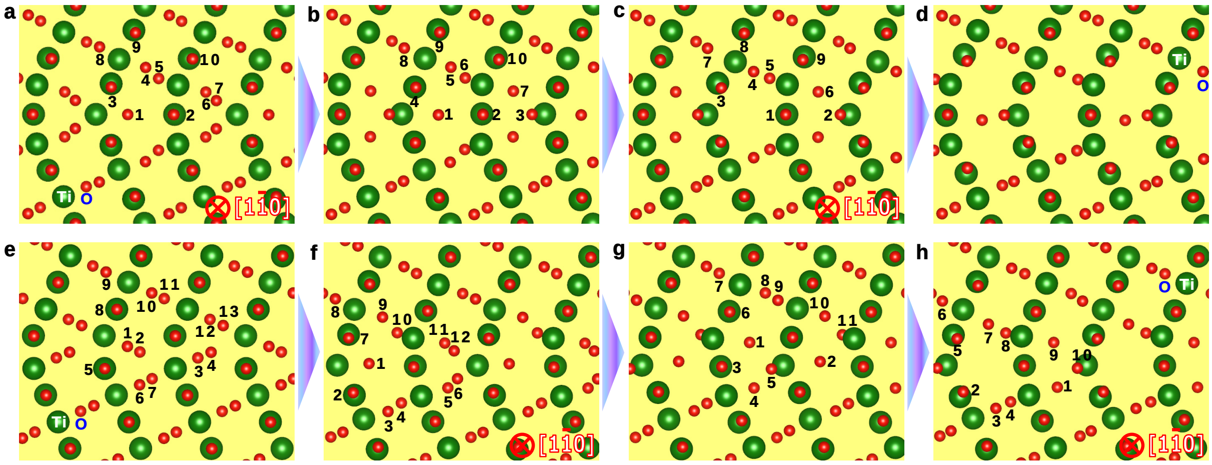

Supplementary Figure 6 | Systematically seeking stable GB. a,e, Two starting models are constructed from the consideration of GB space filling and mirror symmetry: one has single cation layer (**a**) and the other has two (**e**). **b,** Relaxed GB model after introducing an oxygen vacancy to the starting model **a** at all possible positions labeled 1 to 10 in **a**. **c,** Relaxed GB model after introducing an oxygen vacancy to the model **b**. **d,** Relaxed GB model after introducing one oxygen vacancy to the model **c**. **e–h,** A series of GB models obtained by introducing an oxygen vacancy once at a time at the labeled sites, followed by a structural relaxation. The final relaxed model is obtained after introducing an oxygen vacancy at site 9 in **h**, as illustrated in Fig. 3d. Each intermediate model corresponds to the lowest vacancy segregation energy among all the possible sites considered (refer to Supplementary Table 1). The larger balls denote Ti atoms and the smaller ones O atoms.

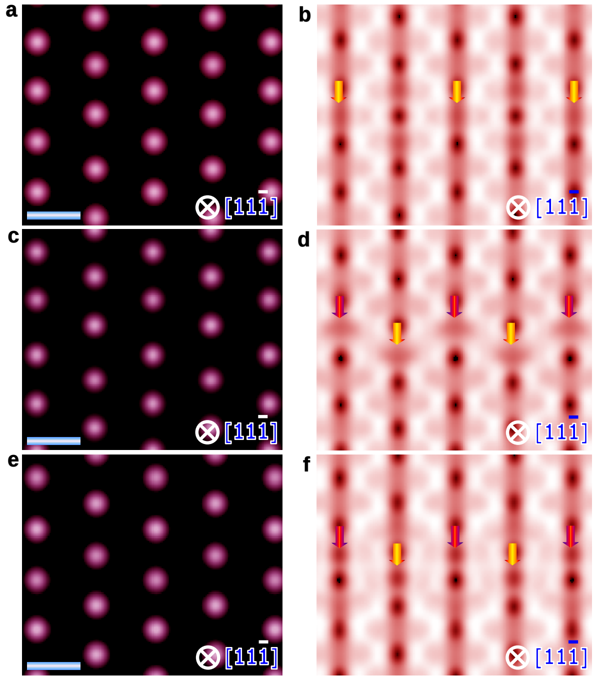

Supplementary Figure 7 | Simulated images. a,b, Simulated HAADF (**a**) and ABF (**b**) STEM images using the determined atomic model of *o*-GB viewed from $[11\bar{1}]$ direction. **c,d,** Simulated HAADF (**c**) and ABF (**d**) STEM images for the *r*-GB viewed from $[11\bar{1}]$ direction. **e,f,** Simulated HAADF (**e**) and ABF (**f**) STEM images for the *v*-GB. Scale bar, 3 Å.

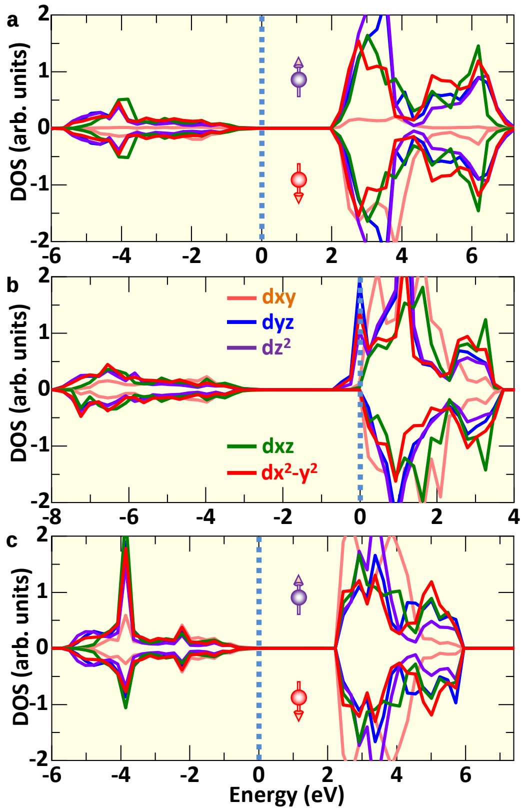

Supplementary Figure 8 | Orbital analysis of GB electronic states. a–c, Partial DOS (PDOS) plots of Ti-3*d* orbitals for the *o*-GB (**a**), *r*-GB (**b**), and *v*-GB (**c**). The Fermi level is set to zero and indicated by a vertical dashed line.

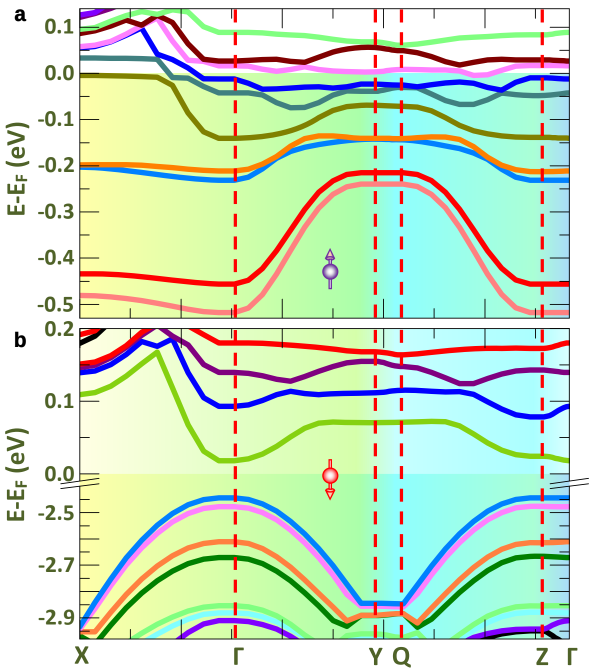

Supplementary Figure 9 | Band structure of the *r*-GB. a,b, Blowup of band structure around Fermi level (E_F) for the *r*-GB along the high-symmetry lines: the majority spin (**a**) and the minority spin (**b**). The E_F is aligned to zero.

Supplementary Table 1. Calculated segregation energies (eV/defect) for the possible oxygen vacancies at the GBs. Two starting models are considered within the GB mirror symmetry: one has a single Ti layer (refer to Supplementary Fig. 4a) and the other two Ti layers (refer to Supplementary Fig. 4e) on the mirror plane. The **a** to **c** and **e** to **h** point to the atomic models illustrated in Supplementary Fig. 4. The sites of the introduced oxygen vacancies at and around GB are labeled on respective atomic model.

**a**	E_{seg}	**b**	E_{seg}	**c**	E_{seg}	**e**	E_{seg}	**f**	E_{seg}	**g**	E_{seg}	**h**	E_{seg}
1	-1.924	1	-2.092	1	-0.279	1	-4.239	1	-1.849	1	-1.886	1	-2.115
2	-0.719	2	-0.260	2	-0.131	2	-6.172	2	-1.230	2	-1.881	2	-0.990
3	-1.563	3	-0.376	3	-1.219	3	-4.239	3	-1.862	3	-0.531	3	-0.153
4	-2.649	4	-0.890	4	-0.014	4	-6.167	4	-0.360	4	-1.885	4	-0.671
5	-0.271	5	-2.088	5	-0.539	5	-1.247	5	-1.685	5	-0.265	5	-1.339
6	-1.591	6	-0.018	6	-1.821	6	-1.676	6	-5.951	6	-1.125	6	-0.216
7	-5.650	7	-1.407	7	-0.170	7	-6.163	7	-1.716	7	-0.005	7	-0.008
8	-0.004	8	-0.006	8	-0.920	8	-1.579	8	-0.014	8	-0.466	8	-0.437
9	-0.924	9	-0.724	9	-0.884	9	-0.080	9	-2.530	9	-0.025	9	-2.116
10	-5.641	10	-1.114			10	-3.131	10	-0.116	10	-1.885	10	-0.148
						11	-0.007	11	-1.682	11	-0.265		
						12	-1.674	12	-5.497				
						13	-6.170						

Supplementary Discussion

Fabrication of pre-designated GBs by bicrystal technique

In general, polycrystalline materials contain various interfaces between grains which can be influenced by numerous factors, such as thermal processing and growth condition. To restrain the degree of freedom associated with grain boundaries (GBs) so as to provide a realistic opportunity to probe every individual GBs, we took advantage of the bicrystal technique to fabricate a model $\Sigma 3(112)[1\bar{1}0]$ GB of TiO_2 by precisely joining two pristine TiO_2 single-crystal blocks of high purity (purity: 99.9%, Shinkosha, Tokyo, Japan) within the bi-crystallographic relationships $(112)[1\bar{1}0]_{upper} \parallel (112)[1\bar{1}0]_{lower}$[1–3], as sketched in Supplementary Fig. 1a. Size of each single-crystal block was set to be 9 × 12 × 5 mm^3, and a bicrystal block of 9 × 12 × 10 mm^3 was eventually acquired. To fabricate bicrystals with the designated orientation relations, the two single crystals were first cut precisely along (112) plane of TiO_2 lattices, followed by one-side grinding and polishing to mirror finish for each crystals with diamond slurry of 0.25 μm. Subsequently, the shiny surfaces of the two single crystals were placed together at 1773 K for 10 h in air. Both heating and cooling rates were set to 300 K/h. Supplementary Fig. 1b shows a real photograph of the final bicrystal where the boundary is indicated by arrows. Several slices with dimensions of 9(12) × 10 × 1.5 mm^3 were cut from the final bicrystal blocks to prepare specimens for the transmission electron microscopy (TEM) and scanning TEM (STEM) observations, which were further thermally treated at different atmosphere.

Atomistic models of grain boundaries

To determine GB atomic models, we construct two starting models within GB mirror

symmetry: one has a cation monolayer (Supplementary Fig. 6a) and the other has a cation bilayer on the GB mirror plane (Supplementary Fig. 6e). These two starting models are O over-stoichiometric at GB from the consideration of space filling (*i.e.* containing enough oxygen atoms at GB), on which one can introduce systematically charge-compensating oxygen vacancies (denoted $V_o^{\bullet\bullet}$ in *Kröger-Vink* notation). Specifically, for the starting model with a cation monolayer, we first consider a total of ten most likely oxygen sites to introduce a vacancy (labeled in Supplementary Fig. 6a) once at a time, and perform a full structural optimization for every model with an oxygen vacancy until the magnitude of force on every atom in the supercell fell below 0.05 eV/Å. The segregation energy (E_{seg}) for an oxygen vacancy at a particular atomic position surrounding the GB in every model is calculated from the difference in total energies of supercells with an oxygen vacancy at GB (E_{GB}) and in bulk (E_{BR}), as expressed by $E_{seg} = (E_{GB} - E_{BR})$[4]. The E_{BR} is calculated by introducing an oxygen atom in the middle (away from GB) of the TiO_2 single-crystal slab in the supercell. With this definition, a negative E_{seg} value indicates that a vacancy prefers to sit at a site at a GB.

For the starting model with a cation monolayer (Supplementary Fig. 6a), we identify that the site 7 is favorable for oxygen vacancy because it shows the lowest E_{seg} (see "**a**" in Supplementary Table 1). Supplementary Fig. 6b shows the corresponding relaxed model, based on which a total of ten possible sites are considered further to introduce an oxygen vacancy once at a time. The GB atomic model with an O vacancy at the site 1 (labeled in Supplementary Fig. 6b) shows the lowest E_{seg} ("**b**" in Supplementary Table 1). Its relaxed model is given in Supplementary Fig. 6c. We further introduce an oxygen vacancy to this

model and find that it favors the site 6 (Supplementary Fig. 6c), that is, the relaxed model (Supplementary Fig. 6d) has the lowest E_{seg} ("**c**" in Supplementary Table 1). We also conduct a similar searching process for the starting model with a cation bilayer. Supplementary Fig. 6e–h illustrates the evolution of models as an O vacancy is gradually introduced. The corresponding segregation energies are listed in Supplementary Table 1. The eventual atomic structure (Fig. 3d) is obtained *via* a relaxation of the GB model with an oxygen vacancy at the site 9 in Supplementary Fig. 6h ("**h**" in Supplementary Table 1). Of all the examined models, we identify three models which match correspondingly the observed images (Fig. 2) of *o*-GB (Fig. 3a), *r*-GB (Fig. 3d) and *v*-GB (Fig. 3g). The relative stability of the three models is shown in Fig. 5a. To provide further support, we simulated HAADF and ABF STEM images using the determined GB atomic models and compared them (Fig. 3, Supplementary Fig. 7) with their experimental counterparts (Fig. 2, Supplementary Fig. 5). A good agreement is found for the two orthogonal projections, thereby validating the application of these models to describe the three GBs.

Since the stoichiometry of every GB configuration can be described in terms of the interfacial excess, the excess of component O with respect to component Ti at the GBs of TiO_2 can be defined as $\Gamma_O = \frac{1}{2A_s}(N_O - N_{Ti}\frac{N_O^{bulk}}{N_{Ti}^{bulk}})$, where A_s is area of the supercell parallel to the boundary (set equal to one in this case), and N_α and N_α^{bulk} are the number of units of component α (α = O or Ti) in the supercell and in the bulk, respectively. By this definition, we calculated Γ_o of the *o*-GB, *v*-GB, and *r*-GB to be 1, 0, and –2, demonstrating that they are locally O over-stoichiometric, stoichiometric, and under-stoichiometric, respectively.

To gain insights into the conditions at which the models are stable, we calculated

Gibbs free energy (γ) of the three GBs as a function of the atomic chemical potentials of constituents (μ_α) using the following expression[5],

$$\gamma(\alpha,q) = \frac{1}{2A}[E(\text{defective},q) - \sum_{\alpha} n_{\alpha}\mu_{\alpha} + qE_{\mathrm{F}}], \quad (1)$$

where E(defective,q) is the energy of a GB supercell with an oxygen vacancy in a charge state q; n_α is the number of α atom; E_F is the electron Fermi energy; A is the interface area. To calculate the γ, we estimated chemical potential of each constituent μ_α by adopting the following equation: $\mu_{Ti} + 2\mu_{O} = E_{Ti(bulk)} + 2E_{O(O2)} + \Delta H_f(TiO_2)$, where E_α denotes the total energy of metal Ti or gaseous O_2, and ΔH_f represents formation enthalpy of TiO_2 (rutile). Here, the μ ($=\mu_O - E_{O(O2)}$) is taken as a variable, spanning a range from ΔH_f(MgO) (Ti-rich or reduction limit) to zero (O-rich or oxidation limit).

Electronic structure of the GBs

To resolve orbital contribution, we calculate partial density of states (PDOS) of the three GBs using their determined atomic geometries, as shown in Supplementary Fig. 8. All the five Ti 3d orbitals almost contribute equally to the valence-band (VB) minima, irrespective of the GB species. The conduction-band (CB) minima for the three species of GBs, however, differ remarkably with one another: the d_{xy} orbital is suppressed heavily in spin-majority channel in the o-GB case (Supplementary Fig. 8a), while all five 3d orbitals contribute to CB in the v-GB case (Supplementary Fig. 8c). The contribution to CB at E_F in the r-GB case originates predominantly from d_{yz}, $d_{x^2-y^2}$, and d_{z^2} orbital in spin-majority channel as revealed in the charge-density isosurface (Fig. 5e). However, there is no state at E_F at all in spin-minority channel, confirming a ferromagnetic alignment of spins for

the *r*-GB. The differences offer unequivocal evidence that local structural transformation can give rise to an orbital modification at the GB, highlighting the relevance in probing GB transformations at the atomic scale.

To shed more light on the electronic nature of the lowest-lying CB for the *r*-GB, we calculated band structure, as shown in Supplementary Fig. 9. Like what was seen in the PDOS plot (Supplementary Fig. 8b), a ferromagnetic alignment of spins is preferred: the Ti 3*d* orbitals are filled around E_F in spin-majority channel as there appear states cross E_F (Supplementary Fig. 9a), while they are empty in spin-minority channel, opening a band gap (Supplementary Fig. 9b). The band structure along the Γ-Z high-symmetry line in the spin-majority channel shows little dispersion. Importantly, the two lowest-lying bands display no discernible dispersion along the Γ-X, while a strong dispersion along the Γ-Y, indicative of a spin-polarized quasi-1D dispersion behavior for the lowest-lying bands of *r*-GB[6]. A total of eight bands cross E_F and contribute to Fermi surface.

Supplementary References

1. Sato, Y., Yamamoto, T. & Ikuhara, Y. Atomic structures and electrical properties of ZnO grain boundaries. *J. Am. Ceram. Soc.* **90,** 337−357 (2007).
2. Nakamura, A. *et al.* Structure and configuration of boundary dislocations on low angle tilt grain boundaries in alumina. *Mater. Trans.* **50,** 1008−1014 (2009).
3. Choi, S. -Y. *et al*. Dislocation structures of low-angle boundaries in Nb-doped $SrTiO_3$ bicrystals. *J. Mater. Sci*. **41,** 2621−2625 (2006).
4. Körner, W. & Elsässer, C. First-principles density functional study of dopant elements at grain boundaries in ZnO. *Phys. Rev. B* **81,** 085324 (2010).

5. Tanaka, I. *et al*. Identification of ultradilute dopants in ceramics. *Nat. Mater*. **2,** 541−545 (2003).
6. Wang, Z. C. *et al*. Spontaneous structural distortion and quasi-one-dimensional quantum confinement in a single-phase compound. *Adv. Mater*. **25,** 218−222 (2013).
